# The incredible shrinking dewlap: signal size, skin elasticity, and mechanical design in the green anole lizard (*Anolis carolinensis*)

**DOI:** 10.1002/ece3.1690

**Published:** 2015-09-19

**Authors:** Simon P. Lailvaux, Jack Leifer, Bonnie K. Kircher, Michele A. Johnson

**Affiliations:** ^1^ Department of Biological SciencesUniversity of New Orleans2000 Lakeshore DriveNew OrleansLouisiana70148; ^2^ Department of Engineering ScienceTrinity UniversityOne Trinity PlaceSan AntonioTexas78212; ^3^ Department of BiologyTrinity UniversityOne Trinity PlaceSan AntonioTexas78212; ^4^ Department of BiologyUniversity of FloridaGainesvilleFlorida32611

**Keywords:** Dewlap, elasticity, evolution, signaling

## Abstract

The expression of male secondary sexual traits can be dynamic, changing size, shape, color, or structure over the course of different seasons. However, the factors underlying such changes are poorly understood. In male *Anolis carolinensis* lizards, a morphological secondary sexual signal called the dewlap changes size seasonally within individuals. Here, we test the hypothesis that seasonal changes in male dewlap size are driven by increased use and extension of the dewlap in spring and summer, when males are breeding, relative to the winter and fall. We captured male green anole lizards prior to the onset of breeding and constrained the dewlap in half of them such that it could not be extended. We then measured dewlap area in the spring, summer, and winter, and dewlap skin and belly skin elasticity in summer and winter. Dewlaps in unconstrained males increase in area from spring to summer and then shrink in the winter, whereas the dewlaps of constrained males consistently shrink from spring to winter. Dewlap skin is significantly more elastic than belly skin, and skin overall is more elastic in the summer relative to winter. These results show that seasonal changes in dewlap size are a function of skin elasticity and display frequency, and suggest that the mechanical properties of signaling structures can have important implications for signal evolution and design.

## Introduction

The evolution and expression of male secondary sexual traits is affected by a variety of factors (Andersson 1994). In some cases, male traits are shaped by female preferences for trait size, color, or shape within limits imposed by the signaling environment, whereas in others, male traits may be influenced primarily by functional or signaling requirements in the context of male combat. The selective contexts of both female choice and male combat may also combine with the various costs of signal expression and maintenance to affect the structure, performance, and expression of male secondary sexual traits in complex ways (Berglund et al. 1996; Dennenmoser and Christy 2013). In addition to these external selection pressures, trait expression may also be affected by internal factors (Wagner and Schwenk 2000) such as those relating the trait to other components of the multivariate organismal phenotype (Emlen 2001; Faivre et al. 2003; Badyaev 2004), or to individual condition and resource availability (Rowe and Houle 1996; Nijhout and Emlen 1998; Tomkins et al. 2004). Both external and internal pressures may fluctuate over time (Bussiere et al. 2008; Kasumovic et al. 2008; Bell 2010), and consequently, the sustained expression of sexually selected traits at high levels may not be optimal. Indeed, previous studies have shown that the expression of such traits can be dynamic and/or cyclical, with clear temporal fluctuations in signal strength or size (Andersson 1994; Hegyi et al. 2007), and in some cases the absolute presence or absence of a signal. For example, male deer will often shed their antlers entirely during the nonbreeding season, only to regrow them again annually in time for the rut (Chapman and Chapman 1997; Ciuti and Apollonio 2011).

The proximate mechanisms underlying dynamic signal expression have received relatively little attention. In the case of behavioral display or acoustic signals, for example, where signal modulation or display effort might be either under individual control or directly and physiologically linked to the pool of available resources to fuel such effort (e.g., Hunt et al. 2004), a specialized mechanism may be self‐evident or unnecessary. However, multiple processes could be involved in the expression and reduction of morphological traits, in particular. The shedding and regrowth of traits such as antlers is likely to be energetically expensive, and life‐history theory tells us that resources allocated to this process would be unavailable for allocation to other fitness‐enhancing traits (James 1974; Van Noordwijk and Dejong 1986). Continued and repeated growth and reduction of traits therefore appears to be an expensive mechanism for dynamic signal expression, unless the resources allocated to such expression could be recovered during trait recrudescence. Another possibility is that the change in shape or size of such traits might be facilitated by the mechanical properties of the morphological structure itself. For example, elasticity in the structural elements that make up a signal could allow that signal to change size or shape in response to specific external or internal conditions without obligatory expenditure of additional resources. Such a mechanism might be favoured relative to energetically costly alternatives such as growth and recrudescence. However, evidence of signal changes that are rooted in the material properties of the signal itself would also raise the possibility that such changes could be nonadaptive consequences of mechanical design, wear, and/or use, depending on the nature of the signal and the way in which it is employed. Thus far, few studies have collected the types of data required to address these issues for any signaling structure.

Male green anole (*Anolis carolinensis*) lizards show dynamic expression in the maximum extended size of their secondary sexual signal, the dewlap, or throat fan. The dewlap is an area of pigmented skin on the underside of the throat supported along the outer edge by the thin, paired second ceratobranchial cartilage. Activation of the paired ceratohyoid muscles at the front of the hyoid apparatus causes the ceratobranchials to extend forward, away from the body, unfurling the dewlap and stretching it out (Bels 1990; Johnson and Wade 2010). Male green anoles extend their dewlaps in combination with headbobs and pushups to form stereotyped sequences of visual displays that are used in a variety of ecological contexts, including courtship displays to females and aggressive displays to other males (Decourcy and Jenssen 1994). These displays have been extensively studied in green anoles (e.g., Jenssen et al. 2000; Lovern and Jenssen 2003; Bloch and Irschick 2006; Edwards and Lailvaux 2012) and occur with significantly higher frequency in the breeding season compared to the nonbreeding season (Jenssen et al. 1995, 2001). Irschick et al. (2006) observed that male green anole dewlaps change size between the breeding and nonbreeding seasons in nature, being larger in the summer when anoles are actively breeding as compared to in the spring and winter. In that same study, Irschick et al. (2006) replicated this phenomenon in the laboratory, showing that it is the individual dewlaps which change size plastically. This finding is intriguing because relative dewlap size has been linked to relative bite force in male *A. carolinensis* (Vanhooydonck et al. 2005a), and bite force itself was also found by Irschick et al. (2006) to vary in concert with dewlap size. One possible explanation for this phenomenon is that males may be allocating a larger proportion of acquired energetic resources toward dewlap expression during the breeding season when they are displaying more frequently relative to the nonbreeding season, resulting in dewlap growth and subsequent shrinkage as animals transition in and out of the breeding period; however, a subsequent dietary‐restriction experiment showed that dewlap size is unaffected by resource availability in growing *A. carolinensis* males, although bite force does decline under low resource conditions (Lailvaux et al. 2012). An alternative hypothesis, which thus far has not been tested, is that the observed seasonal change in individual dewlap size is attributable to the elastic nature of the dewlap skin itself (Irschick et al. 2006). Under this scenario, increased extension frequency during the breeding season causes the dewlap to stretch beyond its initial size seen in the prebreeding seasons when displays are demonstrably less frequent and then to return to that initial size in the postbreeding season when dewlapping frequency is again reduced. This notion is plausible given that elasticity is a mechanical property of vertebrate skin (Spearman 1973), although relatively little is known in this regard about reptile skin specifically (but see Bauer et al. 1989; Klein et al. 2010).

We tested this elasticity hypothesis by comparing changes in dewlap size over the course of a breeding season in adult males that either had mechanically constrained or unconstrained dewlaps. Specifically, we predicted that dewlap size would increase in unconstrained individuals during the period from spring to summer when the males are actively displaying and then shrink in the fall and winter as display activity decreased, whereas dewlap size should be unchanged in males that were prevented from displaying the dewlap at all. We also develop and implement a novel technique for measuring elasticity in skin samples and test the prediction that dewlap skin is significantly more elastic (i.e., exhibits a lower elastic modulus, *E*) than nondewlap skin sampled from the belly. Finally, we compare skin elasticity across the breeding and postbreeding seasons to test the prediction that dewlaps will be more elastic in the summer relative to the winter when dewlaps shrink.

## Methods

### Lizard housing and treatment

All procedures were approved by the University of New Orleans Institutional Animal Care Committee protocol # UNO‐11‐005 and by Trinity University Animal Use Protocols #81809‐MJ1 and #050213‐MAJ2. We collected 35 adult male *A. carolinensis* lizards from a single natural population in Orleans parish, New Orleans, in March 2012 and brought them to the laboratory at the University of New Orleans. We housed lizards individually in 30 × 16 × 16 cm cages lined with cypress mulch and with single 30 × 0.5 cm perches oriented toward uniform 75W lightbulbs, providing opportunities for basking. All cages were located in a room set at 25°C on a 12‐L:12‐D photoperiod as in Irschick et al. (2006) and Lailvaux et al. (2012). We allocated lizards randomly to one of two treatments. We constrained the dewlaps of the lizards in the first treatment (*n* = 17) by lightly tying a piece of dental floss around their necks, which allowed lizards to headbob and swallow food normally, but prevented them from extending their dewlaps. In the second treatment (*n* = 18), we left the lizards unconstrained and allowed them to extend their dewlaps freely and at will. Consistent with previous studies (Irschick et al. 2006; Lailvaux et al. 2012), we covered the sides of each cage with dark paper to prevent the lizards from seeing each other (although lizards do nonetheless perform undirected displays under these conditions even without visual stimuli). The positions of the cages in the room were randomized on a weekly basis to eliminate potential location effects on individual behavior.

### Experimental design

We maintained lizards in the laboratory from March–December. We measured each individual for dewlap size, bite force, mass, and SVL upon capture and then remeasured these same variables in July. Following remeasurement, we selected half of the lizards from each treatment at random and sacrificed them using a sodium Pentobarbital (1.95 mg diluted 1:10 in sterile water) given IC (Ascher et al. 2012). These individuals constituted the summer sample for skin elasticity measures (see below). We maintained the remaining 15 lizards in the laboratory until December, when we measured them a final time before sacrificing them and taking skin samples for the winter skin elasticity measures.

### Measurement of morphology, dewlap size, and bite force

For each lizard, we measured the following variables (consistent with previous studies considering dewlap plasticity): mass, SVL (measured from the tip of the snout to the cloaca), bite force, and dewlap area. We measured bite force because it has been shown to fluctuate seasonally as well in this species (Irschick et al. 2006), and we wanted to ensure that our dewlap treatment did not alter this pattern. As in (Irschick et al. 2006; Lailvaux et al. 2012), we measured dewlap area by photographing the dewlap, extended forwards by gripping the base of the second ceratobranchial with soft forceps, against a 1 cm × 1 cm grid using a Canon A610 Powershot camera mounted on a tripod. We then digitized the dewlap area from the photographs using tpsDig v 1.3.1. This method yields repeatable dewlap area results in anoles, including *A. carolinensis* (Vanhooydonck et al. 2005a,b; Irschick et al. 2006; Lailvaux and Irschick 2007; Lailvaux et al. 2012).

We measured bite force using standard methods. Briefly, lizards were induced to bite forcefully on the free ends of bite plates connected to an isometric Kistler type 9023 force transducer (Kistler, Winterthur, Switzerland) and recorded the resultant force readout from a type 5058a Kistler charge amplifier (see detailed descriptions in Herrel et al. 1999, 2001). Consistent with standard performance methodology (Losos et al. 2002; Adolph and Pickering 2008), we measured bite force five times per individual, with an hour's rest between measures, and retained the highest force measured for analyses. We placed lizards inside an incubator set at 32°C for an hour prior to and in between bite force measures as in previous studies of *A. carolinensis* bite force (Lailvaux et al. 2004, 2012; Irschick et al. 2005, 2006; Vanhooydonck et al. 2005a,b; Husak et al. 2007). We removed dewlap constraints while measuring bite force.

### Measurement of skin elasticity

**Figure 1:**
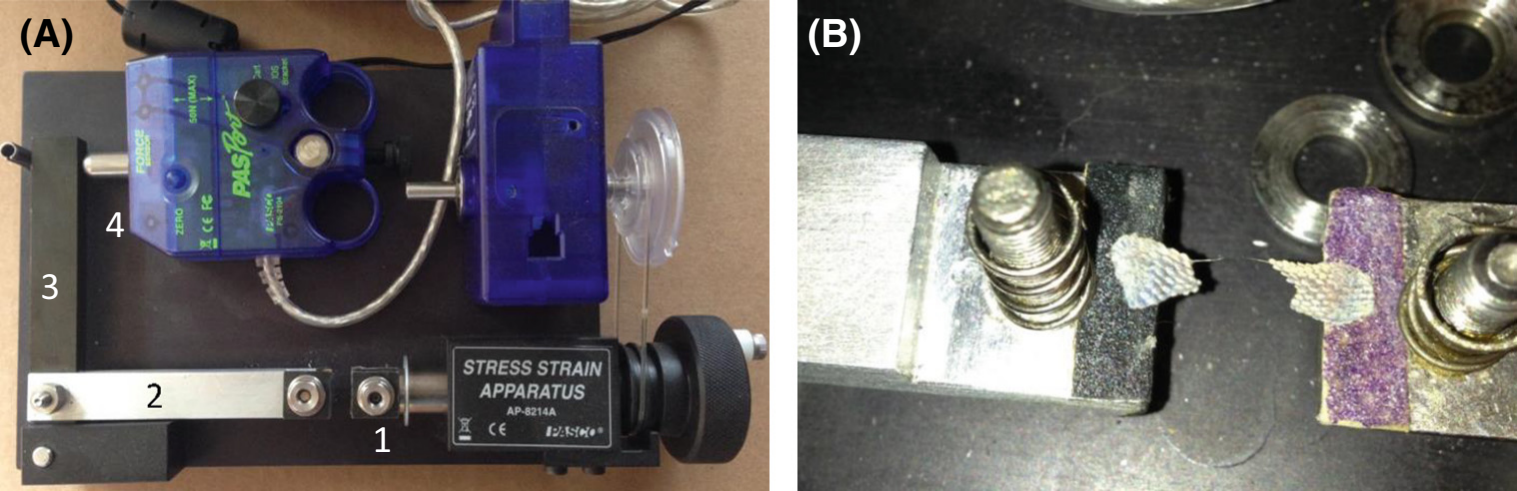
(A) Apparatus for measuring skin elasticity. Tensile force developed in the sample held between the clamps as [1] is displaced to the right is transferred through [2] and the pivoting bar [3] to the force sensor [4]. (B) Detail of [1] showing skin sample on clamps (with the upper clamp surfaces removed). Note that this is a photograph of a sample after it was tested to destruction.

Immediately upon euthanasia, we prepared and trimmed two dewlap and two belly skin samples per animal for testing to mechanical failure. One sample from each skin type was flash‐frozen on dry ice and stored at −80°C until cryosectioning at 20 *μ*m. From these sections, we used ImageJ to measure the thickness of each skin sample (*t* in Equation (2) below) at 100× magnification. The other two samples of each skin type were stored in 70% ethanol at −20°C for use in elasticity measurements. We modified a PASCO stress–strain apparatus (AP‐8214A, PASCO Scientific, Roseville, CA) to measure the force‐deflection behavior of each of these skin samples as they were stretched (Fig. 1A). We placed the two ends of a cut rectangular sample on to the milled flats (i.e., bottom clamp surfaces) of the apparatus, taking care to ensure that each flat held at least 3 mm of skin (Fig. 1B). The section located between the flats made up the test section. Once the sample was on the flats, we adjusted the testing apparatus so that the gap between the two flats was between 3 and 6 mm (depending on the length of the skin sample) and verified values of the initial gap distance and the sample width using digital callipers. We then placed the top pieces onto each clamp and tightened the nuts thereon using a digital torque wrench. Spring compression occurred as the nut located on the top of each clamp was tightened against the spring, allowing the clamping force to be precisely adjusted. Using a digital torque driver to tighten the nut to a set value, we ensured that the clamping force applied to the skin sample was sufficient to prevent slippage, but below a level that would crush the skin. Over the course of the measurements, this nut tightening torque varied between 0.113 N‐m (for the thicker samples) and 0.17 N‐m (for thinner ones). Higher torques were indicative of smaller gaps between the upper and lower clamp surfaces, as smaller gaps required more spring compression, which increased the torque required to rotate the nut against the top of the clamp.

We configured a PASCO Explorer GLX datalogger to plot force as a function of applied displacement. Given the decidedly linear behavior of the skin elasticity curve in the region of skin failure, we determined the elastic constant of skin (*E*) using basic linear expression of Hooke's Law:

(1) E=Lk/A

where *L* is the length of the sample between the flats (active section), *A* is the cross‐sectional area of the sample (product of width and thickness)

(2) A=wt

and *k* is the spring constant that relates the applied force (*F*) to the sample deflection (*δ*) in a specific sample:

(3) k=F/δ

For each sample tested, we obtained a force‐deflection curve that allowed the sample‐specific spring constant (*k*) to be determined. We then combined each spring constant with the corresponding sample geometry (i.e., *L* and *A*) to calculate the linear elastic constant (*E*) of the skin based on the engineering stress placed upon it. We used the average of the two belly samples and the average of the two dewlap samples per individual as our measure of belly and dewlap elasticity, respectively. However, our results are quantitative extremely similar and qualitatively identical if the maximum *E* values per individual are analyzed instead.

### Statistical analyses

To test for differences in changes in dewlap area, bite force, SVL, and mass between dewlap constrained and unconstrained males from spring to summer and summer to winter, we used a general linear mixed‐model (GLMM) with time and dewlap treatment as fixed factors and individual as a random factor (to account for repeated measures) implemented using the *lme* function of the *nlme* package (Pinheiro et al. 2013) for R v. 3.1.0 (R Core Development Team 2014). SVL was log10‐transformed throughout to meet modeling assumptions. We included log10 SVL as a covariate in the models for dewlap and bite force to compare those curves independent of body size. We coded individuals that were missing data for the final time period (i.e., those individuals sacrificed for skin elasticity measurement in the summer) as NA in the R datafile. The GLMM handles these missing data better than alternative types of analyses such as repeated‐measures ANOVA. We used a GLMM with individual as a random factor to test for fixed effects of season, skin type, and dewlap constraint, as well as all possible interactions among these factors, on the transformed elastic modulus, (*E*)^0.14^ (transformation value determined by Box–Cox transformation using the MASS package in R). Model simplification and selection in all cases were based on deletion tests to determine the minimum adequate model (Crawley 2003), and models with and without specific terms were compared using AIC values and log‐likelihood ratio tests. For each analysis, we used maximum likelihood to fit the initial models and for model simplification. Once the minimum adequate models were determined, they were refit using REML.

## Results

**Figure 2:**
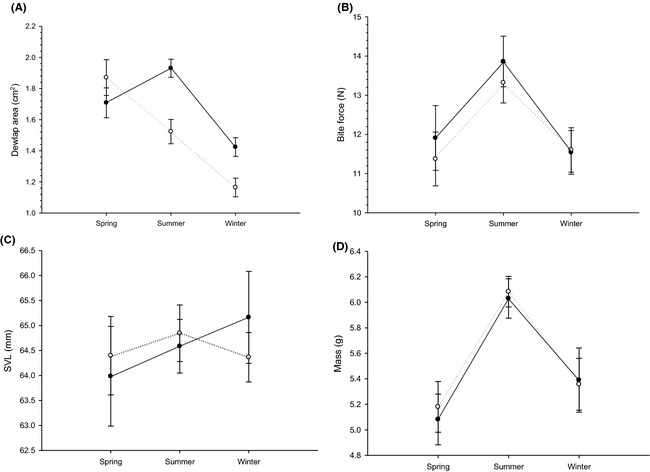
Seasonal changes in (A) dewlap area, (B) bite force, (C) SVL, and (D) mass in dewlap constrained (open circles, dotted lines) and unconstrained (filled circles, solid lines) adult *A. carolinensis* males. Note that sample sizes for winter are half that of the spring and summer samples (see Methods). Error bars represent ±1 SE.

The GLMM for dewlap area retained the full model, including an interaction effect between dewlap treatment and time (Table 1; AIC = 72.42, no. parameters = 4). The dewlaps of males from the constrained and unconstrained treatments therefore followed different trajectories of size change. Indeed, whereas the overall dewlap areas of the unconstrained males mirrored the previous results of Irschick et al. (2006), increasing from spring to summer and then sharply decreasing from summer to winter, the dewlaps of males from the constrained treatment shrank consistently from spring to winter (Fig. 2A). By contrast, none of the minimum models for the other measured traits retained either the interaction between time and dewlap treatment or the lone effect of dewlap treatment as factors (Table 1; bite force AIC = 351.52, no. parameters=2; svl AIC = −436.6, no. parameters = 1; mass AIC = 183.62, no. parameters = 1). In both treatments, bite force increased from spring to summer and subsequently decreased from summer to winter (Fig. 2B), again consistent with the trends reported by Irschick et al. (2006). Importantly, the significant changes in mass over the course of the experiment (Table 1) are indistinguishable between the constrained and unconstrained dewlap treatments (Fig. 2D), strongly suggesting that the dewlap constraint did not interfere with feeding or swallowing in the treatment individuals, and lending further support to the findings of Lailvaux et al. (2012) that changes in dewlap size are unrelated to resource acquisition.

**Table 1:** Best‐fitting GLMMs describing change in dewlap size, bite force, SVL, and mass in dewlap constrained and unconstrained *A. carolinensis* males in the laboratory from spring to winter. The baseline category for time is spring, and for treatment is the constrained dewlap. The reported values therefore give estimated change in the respective dependent variables between the baseline category and the category named in the table

Model term	Coefficient	SE	Traits	Coefficient	SE
Dewlap area			Bite force		
Intercept	−13.19	3.5	Intercept	−142.2	23.19
log10 (SVL)	8.313	1.933	log10 (SVL)	85.149	12.83
Time (summer)	−0.355	0.082	Time (summer)	1.544	0.32
Time (winter)	−0.73	0.114	Time (winter)	−0.107	0.44
Treatment (control)	−0.109	0.106			
Time (summer):Treat (control)	0.538	0.114			
Time (winter):Treat(control)	0.359	0.158			
log10 (SVL)			Mass		
Intercept	1.81	0.003	Intercept	5.13	0.12
Time (summer)	0.004	0.002	Time (summer)	0.91	0.12
Time (winter)	0.007	0.003	Time (winter)	0.35	0.16

**Figure 3:**
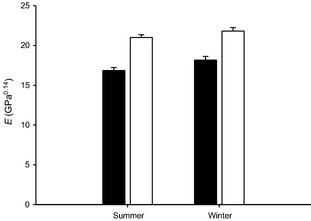
Seasonal changes in transformed elastic constant, *E*, representing resistance to stretch, in dewlap skin (filled bars) relative to belly skin (open bars) in adult *A. carolinensis* males in summer (*n* = 16) and winter(*n* = 15). Error bars represent ±1 SE.

We found an overall effect of time on skin elasticity, with the elastic modulus of skin being significantly lower (i.e., less resistant to stretch) in summer relative to winter (Table 2). We also found an effect of skin type on elasticity (Table 2), and indeed, dewlap skin consistently offered lower resistance to stretching compared to belly skin (Fig. 3). However, we found no effect of treatment on elasticity, suggesting that constraining the dewlaps and preventing them from being extended did not affect the elastic constant of dewlap skin (Table 2). Furthermore, the interactions between treatment and time, treatment and skin type, and the three‐way interaction between treatment, skin type, and time were all excluded from the final model (Table 2, AIC = 251.35, no. parameters = 2). Thus, despite clear changes in the elasticity of *A. carolinensis* skin overall from summer to winter, and the increased elasticity of dewlap skin relative to belly skin, belly skin and dewlap skin exhibited equivalent elastic responses to time and dewlap constraint.

**Table 2:** Best‐fitting model describing the variation in elastic constant (E)^0.14^ by time, treatment, and skin type in male *A. carolinensis*. The baseline category for time is summer, and for skin type is dewlap. The reported values therefore give estimated change in (elastic constant)^0.14^ between the baseline category and the category named in the table

Model term	Coefficient	SE
Intercept	16.978	0.352
Time (winter)	1.056	0.414
Skin type (Stomach)	3.918	0.396

## Discussion

Dynamic expression of secondary sexual traits at the level of the individual has typically been attributed to condition‐dependent phenotypic plasticity that reflects some aspect of that individual's internal physiological state (e.g., Faivre et al. 2003; but see Badyaev 2004). However, an alternative explanation, particularly for morphological traits whose shape or size are altered during display, is that such changes in expression might be a consequence of mechanical design (Thompson 1942) coupled with differences in use. Here, we show that seasonal changes in individual dewlap size in male *A. carolinensis* lizards are indeed a likely consequence of mechanical design, specifically skin elasticity, in conjunction with behavioral use. Our first prediction, that the dewlaps of unconstrained males would increase in size in summer relative to spring and then decrease in winter whereas those of constrained males would remain the same size throughout, was partially supported. We did indeed see the predicted increase and subsequent decrease in dewlap area in the unconstrained male dewlaps (Fig. 2A), consistent with the previous results of Irschick et al. (Irschick et al. 2006). However, the dewlaps of the constrained males did not remain constant; instead, the constrained male dewlaps consistently *decreased* in area over the course of the year, shrinking almost 40% from their initial size in the spring, and ultimately attaining a considerably smaller size in the winter than those of the unconstrained males at the same time of year (Fig. 2A). The fact that the summer peak in unconstrained dewlap size coincides with periods of peak dewlapping and display activity in *A. carolinenisis* (Decourcy and Jenssen 1994; Jenssen et al. 1995), coupled with the clear lack of such a peak in constrained male dewlaps, strongly suggests that it is this dewlapping activity that drives the observed increase in dewlap size, rather than any intrinsic factor such as condition (Lailvaux et al. 2012). However, our results also imply that this effect of dewlapping behavior on dewlap size goes even further: In addition to dewlapping activity apparently increasing dewlap size, the lack of this activity also appears to result in dewlap shrinkage, as illustrated by the size trajectory of the constrained male dewlaps (Fig. 1A). It may be that the level of baseline male displays observed throughout the nonbreeding seasons (Jenssen et al. 1995, 2001) is an important determinant of baseline dewlap size in nonreproductive contexts. Dewlap size therefore appears to ultimately be a function and expression of frequency of dewlap use, in addition to other relevant genetic and environmental factors.

If changes in dewlap size are enabled by skin elasticity, then we also predicted that dewlap skin would prove to be significantly more elastic than nondewlap skin. Our measurements of the elastic modulus (*E*) of dewlap skin samples versus samples of belly skin support this prediction, as *E* (and hence the resistance to stretching) of dewlap skin was indeed significantly lower than that of belly skin in both the summer and the winter sampling periods (Fig. 3). Dewlap skin therefore appears predisposed to stretch more than nondewlap skin, although whether this elasticity is a consequence of past selection for low resistance to stretch in this region or an artifact of continual and repeated extension of the dewlap itself over an animal's lifetime is not apparent from the current dataset. Our finding in this study that dewlap constraint had no effect on the elasticity of dewlap skin may be interpreted as support for the former explanation, although it could be that the time scale of the current experiment was simply too short to reveal significant effects of dewlap stretching on the *E* of dewlap skin.

Our final prediction, that dewlap skin should be significantly more elastic in the summer relative to the winter, was also supported. However, the interaction between time and skin type was not retained in the final elasticity model, which implies that skin *overall* is more elastic during the breeding season, not just dewlap skin specifically. Changes in frequency of dewlap extension cannot explain changes in the elasticity of belly skin, and the underlying mechanism by which this change occurs should therefore be a physiologically general one. Although our current dataset does not provide any insight into this mechanism, potential explanations include changes in hormone profiles between the breeding and nonbreeding seasons, and an aging effect. We consider the latter to be unlikely; although aging is associated with a decrease in skin elasticity in vertebrates due to loss of collagen fibers (e.g., Calleja‐Agius et al. 2007), such effects are very unlikely to manifest over the short duration of the current study. Furthermore, the variation in size, and therefore probably age, within our sample is almost certainly not large enough for this result to have been driven by responses of different age classes within the sample (Fig. 2C). The other explanation, which is that seasonal changes skin elasticity are related to seasonal changes in circulating levels of hormones such as testosterone, is altogether more plausible. Skin is a steroidogenic tissue which metabolizes and responds to sex hormones (Giacomoni et al. 2009), and studies have shown changes in epidermal thickness and elasticity in response to topical steroid application in humans, albeit in the opposite direction to our finding here (Köhn 2006). Commencement of breeding in anoles is associated with a spike in testosterone in males, and cessation of breeding with a decline in circulating testosterone (Tokarz et al. 1998), and this increase in testosterone is itself associated with a suite of behavioral and physiological changes in male green anoles (Husak et al. 2007, 2009). Whether an effect on skin elasticity is one of those changes would be an intriguing topic for future study.

Despite a possibly important relationship between relative dewlap size and relative bite force in several anole species, including *A. carolinensis* (Vanhooydonck et al. 2005a,b; Lailvaux and Irschick 2007), studies have consistently failed to find effects of altering dewlap extension ability or dewlap size on anole ecology in any species (e.g., Tokarz 2002; Tokarz et al. 2003; Henningsen and Irschick 2012). Our finding here that fluctuations in individual dewlap size are a consequence of mechanical design raise additional questions as to the adaptive utility, if any, of the size changes observed in *A. carolinensis* in nature (Thompson 1942). Although we found that dewlap skin is significantly more elastic than belly skin, it is unclear whether this property of dewlap skin is an outcome of past selection for increased dewlap elasticity, or if it is merely a consequence of usage (i.e., long‐term and repeated stretching and extension). Alternatively, it may be the case that dewlap skin *is* selected to be more elastic than other areas of the dermis, but that the observed changes in dewlap size are an incidental byproduct of that elasticity. Measurements of dewlap elasticity over an ontogenetic series of male lizards, from juveniles to adult, in tandem with measures of female dewlaps would constitute a useful first test of this notion in the absence of direct measurements of the form and intensity of selection on dewlap elasticity in nature.

Visual sexual signals comprising or consisting of extensible biological material such as skin are taxonomically widespread and occur in a variety of animal species besides anoles, including (but not limited to): dewlaps, throat pouches, or neck frills in the lizard genera *Urosaurus* (Thompson and Moore 1991), *Polychrus*,* Sitana*,* Otycryptus*,* Draco* (Losos 2009), and *Chlamydosaurus* (Shine 1990); gliding surfaces in *Draco* lizards (Hairston 1957); throat pouches in frigate birds (Madsen et al. 2004); tail “fans” in *Triturus* newts (Green 1991); vocal sacs in frogs such as túngara frogs (Rosenthal et al. 2004); and sexual swellings in primates (Domb and Pagel 2001; Higham et al. 2008). In many such cases, the size of the visual component of the signal is thought to hold meaning in terms of individual characteristics that might be relevant to total fitness, yet alternative nonadaptive explanations for signal size based on material properties of those signals are seldom considered. Our results show that the size of such signals may be affected or altered by frequency of use and/or size change, and as such, signal size could in some cases have a significant plastic component which should be taken into account when weighing the adaptive significance of such signals, especially those such as dewlaps that show dynamic changes over time.

In conclusion, we present evidence for an effect of skin elasticity, coupled with changes in behavioral display activity, on dewlap size over the course of a breeding cycle in male *A. carolinensis* lizards. We also show that dewlap skin is significantly more elastic than belly skin, although the ultimate reasons driving this difference are not apparent from our dataset. Although relatively little is known regarding the mechanical properties of reptile skin, there is currently no particular reason to believe that anoles are in any way exceptional with regard to their skin elasticity. Our results indicate that it would be prudent to contemplate nonadaptive alternatives such as mechanical design when considering changes in morphological structures of which skin is a constituent.

## Conflict of Interest

The authors declare no conflict of interest.
